# Folate deficiency in pregnancy and the risk of preterm birth: A nested case-control study

**DOI:** 10.7189/jogh.14.04120

**Published:** 2024-07-12

**Authors:** Verna Mauren Amy Lazar, Sayedur Rahman, Nabidul Haque Chowdhury, Tarik Hasan, Sharmin Akter, Md Shafiqul Islam, Salahuddin Ahmed, Abdullah H Baqui, Rasheda Khanam

**Affiliations:** 1 Department of International Health, Johns Hopkins Bloomberg School of Public Health, Baltimore, Maryland, USA; 2 Department of Women’s and Children’s Health, Uppsala University, Sweden; 3 Projahnmo Research Foundation, Dhaka, Bangladesh; 4 International Center for Diarrhoeal Disease Research, Bangladesh, Mohakhali, Dhaka, Bangladesh

## Abstract

**Background:**

Preterm birth (PTB) and its complications are important public health problems. Its aetiology is multifactorial and involves both modifiable and non-modifiable factors. Among the modifiable risk factors, micronutrient deficiencies, including maternal folate deficiency, are increasingly being studied in PTB. In this study, we estimated the prevalence of folate deficiency during pregnancy and examined its association with PTB among rural Bangladeshi women.

**Methods:**

We conducted a nested case-control study using data from a population-based cohort of 3000 pregnant women who were enrolled between 8 and 19 weeks of gestation following ultrasound confirmation of gestational age. Sociodemographic, epidemiologic, clinical, and pregnancy outcomes data were collected through home visits, while blood samples were collected at enrolment and 24–28 weeks of gestation during pregnancy. We included all women who delivered preterm (defined as live births <37 weeks of gestation) as cases (n = 235) and a random sample of women having a term birth as controls (n = 658). The main exposure was folate concentrations in maternal serum during 24–28 weeks of pregnancy. We categorised women into folate deficient (<3 ng/mL) and not deficient (≥3 ng/mL). We then performed multivariable logistic regression analysis to examine the association between maternal folate levels and PTB, adjusting for relevant covariates.

**Results:**

Thirty-eight per cent of the enrolled pregnant women were folate deficient. Maternal serum folate deficiency was significantly associated with PTB (adjusted OR (aOR) = 1.73; 95% confidence interval (CI) = 1.27–2.36). The risk of PTB was also higher among women who were of short stature (aOR = 1.83; 95% CI = 1.27–2.63), primiparous (aOR = 1.60; 95% CI = 1.15–2.22), and had exposure to passive smoking (aOR = 1.54; 95% CI = 1.02–2.31).

**Conclusions:**

The prevalence of folate deficiency was high among pregnant women in rural Bangladesh, and folate deficiency was significantly associated with an increased risk of preterm birth.

Preterm birth (PTB), defined as the birth of an infant before 37 weeks of gestation, is a significant global public health problem [1,2]. According to the World Health Organization (WHO), nearly 14 million babies were born preterm worldwide in 2020, accounting for more than one premature birth for every 10 babies born [3]. PTB and its complications are among the leading causes of death in children under five years of age, accounting for approximately one million deaths each year worldwide [4–6]. Most PTBs occur in Southeast Asia and sub-Saharan Africa [7–10]. In this context, Bangladesh was ranked sixth among countries with high PTB, with an estimated PTB rate of 16.2 per 1000 live births in 2020, highlighting the need for targeted interventions to address this critical public health challenge [11].

The aetiology of PTB is multifactorial and includes macro-level geographic determinants such as higher rates of infections in LMICs; sociodemographic characteristics, including racial and ethnic factors; and environmental influences such as air pollution [12–14]. Along with these, a host of maternal factors such as maternal infections; macronutrient and micronutrient deficiencies; pregnancy complications; and individual immunologic, genetic determinants also increase the risk of PTB [14–19]. **Figure 1** shows the factors related to PTB and their potential pathways [15–18].

**Figure 1:**
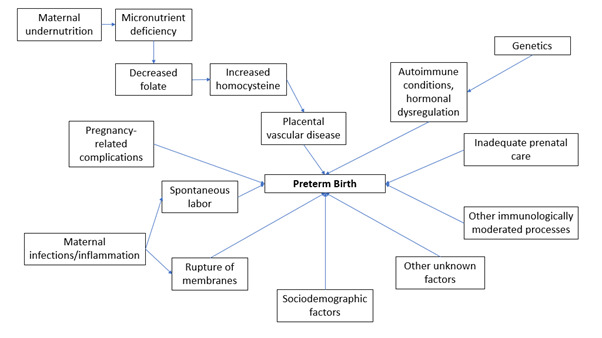
Factors associated with preterm birth and the potential pathways.

Among the nutritional factors, folate deficiency deserves special scrutiny. A study on the global folate status in women of reproductive age reported a >20% prevalence of folate deficiency in low-income countries [20]. Further, inadequate dietary intake and poor absorption or utilisation can lead to folate deficiency, potentially setting the stage for adverse pregnancy outcomes, including PTB [21]. Adequate folate levels in maternal blood are crucial in maintaining general well-being during pregnancy, as well as the growth and development of the foetus [22]. Folate, in particular, is recognised for its role in crucial cellular processes, including DNA synthesis and methylation [21], and the need for adequate folate intake has been outlined in previous studies that aimed to prevent developmental anomalies [23–25]. It is also an important determinant of plasma homocysteine, whereby raised total homocysteine levels in maternal blood and amniotic fluid were found to be associated with increased adverse pregnancy outcomes, including PTB [26]. Independent of homocysteine, folate has also been found to affect maternal placental development and foetal growth through epigenetic changes and antioxidant properties [27].

The current literature on folate deficiency and PTB is mixed; some studies reported an association, while others did not. However, there is a lack of data on the role of folate on PTB from settings where both PTB rates and folate deficiency are high. We designed this study to investigate the relationship between serum folate concentrations and PTB in a setting, taking advantage of a pregnancy biorepository we had established in our rural study site in Bangladesh.

## METHODS

### Study design, setting, and data

We employed a nested case-control study design using data from a population-based cohort of pregnant women and their children, conducted as part of the research initiative known as the Alliance for Maternal and Newborn Health Improvement (AMANHI). The AMANHI study in Bangladesh included the establishment of a biorepository in 3000 pregnant women in two rural sub-districts in the Sylhet district of Bangladesh between 2014 and 2018 [28]. Briefly, trained community health workers (CHWs) with a minimum of 10th grade of schooling made home visits every two months to identify pregnant women by way of a strip-based test. CHWs obtained informed consent from the pregnant women in the local language for a screening ultrasound scan to determine gestational age (GA) and enrolment in the study if the GA was between 8 and 19 weeks. The consent also included follow-up visits and the collection of bio-samples during pregnancy, delivery, and six weeks postpartum. We collected maternal blood and urine samples twice during pregnancy (8–19 weeks and 24–28 weeks or 32–36 weeks of gestation). Apart from collecting biological samples, the CHWs collected detailed sociodemographic, epidemiological, clinical, and pregnancy outcomes data.

### Bio-sample

Trained phlebotomists collected the blood samples at the study clinic. The samples were centrifuged, while serum and plasma samples were aliquoted and preserved at −80°C using standard operating procedures. For this study, serum samples collected between 24 and 28 weeks of gestation of all PTB (<37 weeks of gestation) cases and a sample of controls were tested for serum folate levels.

### Folate assay

Serum folate was measured by a fully automated immunoassay analyser (Cobas e601(Roche Diagnostics GmbH, Mannheim, Germany)) using a commercial kit (Elecsys Folate III (Roche Diagnostics)) according to the manufacturer’s instruction at the Immunobiology, Nutrition, and Toxicology Laboratory of the International Centre for Diarrhoeal Disease Research, Bangladesh (icddr,b). This assay is a competitive electrochemiluminescence protein binding assay that has been standardised against the WHO International Standard NIBSC code: 03/178. Results were automatically calculated via specifically generated calibration curves. Two levels of quality control serum were run in each lot per day to check both accuracy and precision. To ensure the quality of results, the laboratory also participated in the K panel of the College of American Pathogenesis Proficiency Testing Programs.

### Population, cases, and controls

The source population for this study were all women who had the antenatal blood drawn between 24 and 28 weeks of gestation (n = 2287); had at least one antenatal and one postnatal visit by the CHWs and had pregnancy outcome data available (n = 2075); and delivered a live-born singleton baby (n = 1981). We excluded women who delivered stillbirth (n = 78) or twin babies (n = 16). We included all women experiencing preterm delivery (n = 241) and a sample of women who had term births (n = 668).

### Sample size calculation

**Figure 2:**
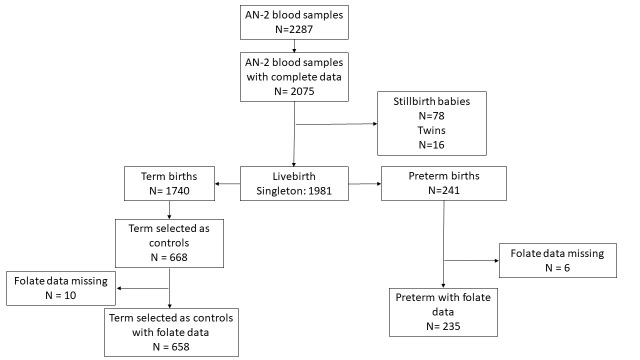
Study flowchart. AN-2 – second antenatal visit.

Using Kelsey’s formula [29] for calculating sample size for an unmatched case-control study, and given a fixed number of cases (n = 241), we determined the required number of controls per case to detect an odds ratio (OR) of 1.3. The parameters for this calculation included a proportion of controls with exposure at 20%; a proportion of cases with exposure at 25%; a two-sided confidence interval (CI) of 95%, and a power of 80%. We selected the assumed OR and exposure rates for controls and cases based on previously published studies [20,30]. The number of controls required per case was 2.8. We randomly selected 668 controls from the available number of controls. We then performed the assessment of folate levels on the cases and controls. Notably, 6 cases and 10 control women lacked available folate measurements and were excluded from the analysis. Therefore, the final analysis included a total of 235 case women and 658 control women (**Figure 2**).

### Measurements

The outcome variable was preterm birth and the main exposure variable was maternal serum folate concentrations measured during 24–28 weeks of gestation.

We initially examined the association between folate concentrations and PTB by dividing women’s folate level into quartiles (lowest quartile (≤2.32 ng/mL), 2nd quartile (2.33–3.83 ng/mL), 3rd quartile (3.84–7.26 ng/mL), and highest quartile (≥7.33ng/mL)). For the final analysis, we categorised folate concentrations into deficient (<3 ng/mL) and not deficient (≥3 ng/mL) based on previously published studies [31,32].

We included several covariates in the analysis and categorised them as follows: maternal age as either less than 30 years or equal to/greater than 30 years; parity into three categories: 0/primiparous, 1–3, and four or more births; maternal and paternal education levels into two groups: 0–5 years of schooling and more than 5 years of schooling. Maternal height was categorised as either below 145 cm or 145 cm and above. The household crowding index was computed by dividing the total number of individuals by number of sleeping rooms. We classified household crowding into two categories: a crowding index of 2 or lower and a crowding index exceeding 2. We applied principal component analysis to create household wealth scores, considering the housing materials and household possessions. We then used these wealth scores to divide the households into wealth tertiles.

### Statistical analysis

Besides examining the association between folate concentrations and PTB, we investigated the association between folate concentrations (categorised as deficient vs not deficient) and a set of covariates: mother's age, education, occupation, maternal height, tobacco consumption, iron-folate supplementation, husband’s education, husband’s occupation, household crowding, and household wealth index. We also examined the association between preterm vs term birth with selected socioeconomic and demographic characteristics of the women. To test for statistical significance, we used the χ^2^ test for homogeneity. We then conducted a logistic regression analysis to estimate the unadjusted ORs and adjusted odds ratios (aORs), along with their corresponding 95% CIs, to identify factors significantly associated with PTB. We estimated the net effect of folate level on PTB after adjusting for covariates. The covariates included in the multivariate logistic regression model were those with a *P*-value <0.05 in the univariate analyses.

We conducted the data analysis using Stata, version 17 (StataCorp LLC, College Station, Texas, USA).

## RESULTS

Regarding the distribution of folate concentrations categorised into quartiles and their relationship with PTB, we observed a trend showing increasing risk of PTB with decreasing folate level; however, compared to women in the highest quartile, the risk of PTB was statistically significantly higher only in women in the lowest quartile (OR = 1.84; 95% CI = 1.22–2.79). The second quartile also showed a higher risk of PTB, but this was not statistically significant. According to the summary category of deficient (<3 ng/mL) and not deficient (≥3 ng/mL), about 38% of women had folate deficiency. The women in the folate-deficient group had a higher risk of PTB (OR = 1.75; 95% CI = 1.30–2.37) compared to the not-deficient group (**Table 1**).

**Table 1:** Association of preterm birth and folate concentrations

	Total (n = 893)	Term (n = 658)	Preterm (n = 235)	OR (95% CI)	*P*-value
**Folate (quartiles)**					
Highest (7.33–23.7)	223 (24.97)	172 (26.14)	51 (21.70)	ref	
3rd quartile (3.84–7.26)	223 (24.97)	179 (27.20)	44 (18.72)	0.83 (0.53–1.30)	0.372
2nd quartile (2.33–3.83)	223 (24.97)	162 (24.62)	61 (25.96)	1.27 (0.83–1.96)	0.270
Lowest (0.63–2.32)	224 (25.08)	145 (22.04)	79 (33.62)	1.84 (1.22–2.79)	0.004
**Folate (two categories)**					
Deficient (<3 ng/ml)	337 (37.74)	225 (34.19)	112 (47.66)	1.75 (1.30–2.37)	<0.001
Not deficient (≥3 ng/ml)	556 (62.26)	433 (65.81)	123 (52.34)	ref	

CI – confidence interval, OR – odds ratio, ref – reference group

In view of the association between maternal serum folate concentrations and selected attributes of both mothers and households, maternal education, chewing tobacco, husband education and occupation, household wealth, and crowding status were significantly associated with folate deficiency (**Table 2**). For the association of preterm vs term birth with selected maternal and household characteristics, maternal height, parity, and exposure to passive smoking were associated with PTB (**Table 3**).

**Table 2:** Distribution of maternal serum folate concentrations by selected characteristics of mothers and households

		Folate concentrations, n (%)		
**Level by factor**	**Total (n = 893)**	**Deficient (n = 337)**	**Not deficient (n = 556)**	***P-*value**
**Maternal age**				0.96
<30 y	785	296 (87.8)	489 (87.9)	
≥30 y	108	41 (12.2)	67 (12.1)	
**Maternal years of education**				<0.001
0–5 y	419	193 (57.3)	226 (40.6)	
>5 y	474	144 (42.7)	330 (59.4)	
**Women's occupation**				0.83
Not employed	873	329 (97.6)	544 (97.8)	
Employed	20	8 (2.4)	12 (2.2)	
**Maternal BMI at baseline**				0.45
Low (<18.5 kg/m^2^)	308	117 (34.7)	191 (34.4)	
Normal (18.5–24.99kg/m^2^)	543	208 (61.7)	335 (60.3)	
High (>25kg/m^2^)	42	12 (3.6)	30 (5.4)	
**Maternal height at baseline**				0.75
<145 cm	170	66 (19.6)	104 (18.7)	
≥145 cm	723	271 (80.4)	452 (81.3)	
**Chewing tobacco**				0.001
No (never, quit pre/during pregnancy)	739	261 (77.4)	478 (86.0)	
Yes (currently sniffing/chewing)	154	76 (22.6)	78 (14.0)	
**Years of education of husband**				<0.001
0–5 y	630	262 (77.7)	368 (66.2)	
>5 y	263	75 (22.3)	188 (33.8)	
**Occupation of the husband**				0.001
Government/private/self	251	74 (22.0)	177 (31.8)	
Daily wage/farming/other/does not work	642	263 (78.0)	379 (68.2)	
**Household crowding index**				0.003
≤2	637	221 (65.6)	416 (74.8)	
>2	256	116 (34.4)	140 (25.2)	
**Household wealth index**				0.001
Poorest	414	182 (54.0)	232 (41.7)	
Middle	191	67 (19.9)	124 (22.3)	
Richest	288	88 (26.1)	200 (36.0)	

BMI – body mass index

**Table 3:** Distribution of infant’s preterm and term status by selected socioeconomic, demographic, and care-seeking characteristics of mothers

Level by factor	Total (n = 893)	Preterm birth (<37 weeks) (n = 235), n (%)	Term birth (≥37 weeks) (n = 658), n (%)	*P*-value
**Folate ng/mL**				<0.001
Not deficient	556	123 (52.3)	433 (65.8)	
Deficient	337	112 (47.7)	225 (34.2)	
**Sex of the baby**				0.39
Male	446	123 (52.3)	323 (49.1)	
Female	447	112 (47.7)	335 (50.9)	
**Maternal age**				0.92
<30 y	785	207 (88.1)	578 (87.8)	
≥30 y	108	28 (11.9)	80 (12.2)	
**Maternal years of education**				0.62
0–5 y	419	107 (45.5)	312 (47.4)	
>5 y	474	128 (54.5)	346 (52.6)	
**Maternal height at baseline**				<0.001
<145 cm	170	62 (26.4)	108 (16.4)	
≥145 cm	723	173 (73.6)	550 (83.6)	
**Maternal BMI**				0.19
Low (<18.5 kg/m^2^)	308	92(39.1)	216(32.8)	
Normal (18.5–24.99kg/m^2^)	543	134(57.0)	409(62.2)	
High(>25kg/m^2^)	42	9(3.8)	33(5.0)	
**Parity**				0.012
0/primi	301	92 (39.1)	209 (31.8)	
1 to 3	502	113 (48.1)	389 (59.1)	
≥4	90	30 (12.8)	60 (9.1)	
**Place of delivery**				0.41
Facility (hospital)	662	170 (72.3)	492 (74.8)	
Home (residence)	231	65 (27.7)	166 (25.2)	
**Delivery conducted by**				0.46
Trained birth attendant	872	228 (97.0)	644 (97.9)	
Untrained birth attendant	21	7 (3.0)	14 (2.1)	
**Iron/folate supplementation**				0.15
No	326	95 (40.4)	231 (35.1)	
Yes	567	140 (59.6)	427 (64.9)	
**Chewing tobacco**				0.19
No (never, quit pre/during pregnancy)	739	188 (80.0)	551 (83.7)	
Yes (currently sniffing/chewing)	154	47 (20.0)	107 (16.3)	
**Passive smoking**				0.028
No	186	37 (15.7)	149 (22.6)	
Yes	707	198 (84.3)	509 (77.4)	
**Years of education of husband**				0.17
0–5 y	630	174 (74.0)	456 (69.3)	
>5 y	263	61 (26.0)	202 (30.7)	
**Occupation of husband**				0.13
Government/private/self	251	57 (24.3)	194 (29.5)	
Daily wage/farming/other/does not work	642	178 (75.7)	464 (70.5)	
**Household crowding index**				0.95
≤2	637	168 (71.5)	469 (71.3)	
>2	256	67 (28.5)	189 (28.7)	
**Wealth index**				0.74
Poorest	414	112 (47.7)	302 (45.9)	
Middle	191	52 (22.1)	139 (21.1)	
Richest	288	71 (30.2)	217 (33.0)	

BMI – body mass index

In the unadjusted logistic regression analysis, in addition to folate deficiency, several risk factors were associated with PTB. For example, women’s height (OR = 1.82; 95% CI = 1.27–2.60), primiparity (OR = 1.51; 95% CI = 1.09–2.09), and exposure to passive smoking during pregnancy (OR = 1.55; 95% CI = 1.04–2.30) showed an increased PTB risk, while iron-folate supplementation was not significantly associated with the risk of preterm birth (**Table 4**).

**Table 4:** Risk of preterm births in folate insufficient mothers after adjusting for covariates

	Preterm birth	
	**Unadjusted OR (95% CI)**	**Adjusted OR (95% CI)**
**Folate concentration**		
Not deficient	ref	ref
Deficient	1.75 (1.29–2.37)	1.73 (1.27–2.36)
**Sex of the baby**		
Boy	ref	
Girl	0.87 (0.65–1.18)	
**Mother's age**		
<30	ref	
>30	0.98 (0.61–1.55)	
**Mother's education**		
0–5 y	ref	
>5 y	1.08 (0.80–1.45)	
Mother's height		
<145cm	1.82 (1.27–2.60)	1.83 (1.27–2.63)
>145cm	ref	ref
**Parity**		
0/primi	1.51 (1.09–2.09)	1.60 (1.15–2.22)
1 to 3	ref	ref
≥4	1.72 (1.05–2.80)	1.58 (0.97–2.58)
**Delivery place**		
Facility	ref	
Home	1.15 (0.82–1.61)	
**Skilled delivery assistance**		
Trained birth attendant	ref	
Untrained	1.41 (0.56–3.54)	
**Iron/folate supplementation**		
No	1.25 (0.92–1.70)	
Yes	ref	
**Tobacco consumption**		
No	ref	
Yes	1.28 (0.87–1.88)	
**Passive smoking**		
No	ref	ref
yes	1.55 (1.04–2.30)	1.54 (1.02–2.31)
**Religion**		
Muslim	ref	
Hindu	0.90 (0.57–1.40)	
**Husband's education**		
0-5 y	1.26 (0.90–1.76)	
>5 y	ref	
**Household crowding**		
≤2	ref	
>2	0.98 (0.71–1.37)	
Wealth index		
Poorest	1.13 (0.80–1.60)	
Middle	1.14 (0.75–1.73)	
Richest	ref	

CI – confidence interval, OR – odds ratio, ref – reference group

In the adjusted logistic regression, maternal serum folate deficiency remained significantly associated with the risk of PTB (aOR = 1.73; 95% CI = 1.27–2.36). Women with a height below 145 cm had a nearly twofold risk of PTB (aOR = 1.83; 95% CI = 1.27–2.63) compared to those with a height of equal to or above 145 cm. Primiparous women also experienced about 1.6 times higher PTB risk (aOR = 1.60; 95% CI = 1.15–2.22) compared to mothers with one to three children. Additionally, women who reported passive smoking had a 1.5 times higher risk of PTB (aOR = 1.54; 95% CI = 1.02–2.31) compared to those who did not report passive smoking (**Table 4**).

## DISCUSSION

We documented a high prevalence of maternal folate deficiency during the second trimester of pregnancy and observed that folate deficiency was significantly associated with the risk of PTB in Bangladeshi women. Aside from folate deficiency, women with short-stature, primiparous women, and those having exposure to passive smoking also had an increased risk of PTB.

The literature on the association between folate deficiency during pregnancy and PTB had conflicting findings [22,27,33–36]. In a cohort study in the Netherlands, Bergen et al. [27] observed that women in the lowest quintile of folate concentrations had twice the risk of spontaneous PTB compared to women in the highest quintile (aOR = 2.17; 95% CI = 1.34–3.57). A prospective cohort study conducted in North Carolina by Siega-Riz and colleagues in 2004 exploring the association of maternal folate status during the second trimester with PTB reported that the lowest tertile had 1.5 times higher odds compared to the highest one (OR = 1.5; 95% CI = 1.0–2.4) [33]. Another cohort study in 313 pregnant women in Pittsburgh, USA found that none of the women were folate deficient (<3 ng/ml), yet women in the highest tertile of folate concentration had an 80% lower risk of spontaneous PTB compared to the lowest tertile [34]. Despite the absence of clinical folate deficiency in the study, the authors observed a negative linear relationship between total folate concentrations and the risk of spontaneous PTB, implying that the relatively higher concentrations of folate may be important in preventing PTB [34]. A case-control study in China found that the lowest-quartile folate concentrations had a higher incidence of preterm births compared to the highest quartile (5.76 vs 2.92), with higher levels of folate being protective against adverse pregnancy outcomes including PTB (aOR = 0.35; 95% CI = 0.25–0.50) [35].

Conversely, a US-based study did not find a significant association between folate deficiency at the time of delivery and PTB after adjusting for maternal age, BMI, marital status, and race [36]. Moreover, a nested case-control study conducted in Australia and New Zealand found no significant association between serum folate, vitamin B12, or homocysteine levels during the second trimester of pregnancy and any adverse pregnancy outcomes [37]. A longitudinal study in Japan looking at the association of folate levels during the first trimester of pregnancy with adverse pregnancy outcomes found no association between mean serum folate levels and preterm birth [38]. The lack of an association observed in these studies might be due to differences in the timing of measurement of folate levels, and possible folate supplementation. For example, the authors of the aforementioned Japan-based study noted that the participants were informed about their folate level and speculated that folate-deficient individuals might have taken folate supplements later in the pregnancy, thus improving their folate status and birth outcomes [38]. These contrasting findings emphasise the contribution of our study, which establishes the link between maternal folate deficiency and the risk of preterm birth, as well as the importance of context-specific research.

The results of randomised controlled trials (RCTs) of folate supplementation are also indecisive, with some studies showing an effect of supplementation in reducing the burden of PTB and others finding no such effect [39,40]. A cluster RCT in rural China found that individuals who received iron-folic acid and those who received multiple micronutrients had a 0.23-week and 0.19-week longer gestation, respectively, compared to those who received placebo [39]. Iron-folic acid supplementation was also associated with a significantly reduced risk of early preterm delivery (<34 weeks), with a risk ratio of 0.50 (95% CI = 0.27–0.94) [39]. However, a systematic review and meta-analysis of five RCTs, two conducted in the UK and three in low-resource settings (Nepal, South Africa, and India), found that folic acid supplementation during pregnancy did not prevent PTB <37 weeks or early PTB <34 weeks [40]. The authors speculated that the lack of effect could be due to delayed start of FA supplementation or that FA supplementation could have been beneficial only if there was folate deficiency, which was not evaluated in the RCTs [40].

Our finding that maternal short stature is associated with PTB was similar to a study in Swedish women [41] and an earlier study conducted in our cohort in Bangladesh [42]. In a study in Missouri, USA, primiparous women had a 1.1-fold higher risk of spontaneous preterm and a 1.3-fold higher risk for medically indicated PTB [43].

However, the studies exploring the association between passive smoking and PTB have had conflicting findings. A study conducted in China found that maternal passive smoking was associated with twofold higher odds of very PTB (<32 weeks) [44]. In the Generation R study (a population-based prospective cohort study in Netherlands), Jaddoe et al. [45] did not find an association between passive smoking and PTB. Potential mechanisms through which passive smoking may influence PTB include its harmful effects on placental development and function through compounds such as nicotine, carbon monoxide and other toxins [44].

Lastly, the mechanism of maternal folate deficiency and PTB is not fully understood. Folate deficiency may cause PTB by elevating homocysteine, along with variants in the methylenetetrahydrofolate reductase gene [21]. Other possible mechanisms may include altered one-carbon metabolism, oxidative stress and altered epigenetic regulation of neurotrophic factor [46]. Bergen et al. [27] proposed that folate deficiency causes PTB through both homocysteine-dependent and homocysteine-independent pathways. Folate acts through an antioxidant mechanism affecting placental implantation and vascular remodelling, crucial for foetal development. Future research should explore the genetic interactions and nutritional status of women to gain a comprehensive understanding of the underlying mechanisms.

Our study has several strengths. The cases and controls were selected from a population-based prospective study. The primary outcome, PTB, was determined through early ultrasound dating before 20 weeks of gestation by professional sonologists, making the classification into term or preterm categories reliable compared with the classification by last menstrual period dates. However, this study also has some limitations. Since it uses a case-control design, it may be susceptible to confounding and selection bias [47]. However, selection biases should be minimal, as we selected all PTB cases of the cohort [48]. Furthermore, we lacked data on several potential risk factors for folate deficiency and PTB, including access to prenatal care, data on dietary intake of folate in pregnant women, and complications during pregnancy. These factors might have confounded the observed relationship. Another limitation of our study is the timing of measurement of folate levels. As the role of maternal folate levels during early pregnancy in embryonic development is well established, future research should consider comprehensive folate assessments across all gestational stages.

## CONCLUSIONS

Our findings are important for low- and middle-income countries such as Bangladesh, as they provide valuable information on the high prevalence of maternal folate deficiency during pregnancy and the associated risk of PTBs, especially because PTB brings significant healthcare costs and adverse outcomes for both mothers and infants. By identifying the relationship between maternal folate deficiency and preterm birth, we highlight a focus area for further research and targeted interventions in Bangladesh and similar settings. Implementation strategies to improve maternal folate status through dietary supplementation, fortified foods, and targeted healthcare interventions may have a significant impact on reducing the burden of preterm birth and its associated complications [49,50].
